# MEK inhibition causes BIM stabilization and increased sensitivity to BCL-2 family member inhibitors in RAS-MAPK-mutated neuroblastoma

**DOI:** 10.3389/fonc.2023.1130034

**Published:** 2023-02-21

**Authors:** Thomas F. Eleveld, Lindy Vernooij, Linda Schild, Bianca Koopmans, Lindy K. Alles, Marli E. Ebus, Rana Dandis, Harm van Tinteren, Huib N. Caron, Jan Koster, Max M. van Noesel, Godelieve A. M. Tytgat, Selma Eising, Rogier Versteeg, M. Emmy M. Dolman, Jan J. Molenaar

**Affiliations:** ^1^ Princess Máxima Center for Pediatric Oncology, Utrecht, Netherlands; ^2^ Department of Oncogenomics, Amsterdam UMC, Amsterdam, Netherlands; ^3^ Hoffmann-La Roche, Basel, Switzerland; ^4^ Children’s Cancer Institute, Lowy Cancer Centre, UNSW Sydney, Kensington, NSW, Australia; ^5^ School of Clinical Medicine, UNSW Medicine & Health, UNSW Sydney, Kensington, NSW, Australia; ^6^ Department of Pharmaceutical sciences, Utrecht University, Utrecht, Netherlands

**Keywords:** MEK, b-cell lymphoma 2 (BCL-2), neuroblastoma, trametinib (PubChem CID: 11707110), navitoclax (ABT-263, PubChem CID: 24978538), venetoclax (ABT-199, PubChem CID: 49846579), synergy, pediatric cancer

## Abstract

**Introduction:**

Mutations affecting the RAS-MAPK pathway occur frequently in relapsed neuroblastoma tumors and are associated with response to MEK inhibition *in vitro*. However, these inhibitors alone do not lead to tumor regression *in vivo*, indicating the need for combination therapy.

**Methods and results:**

*Via* high-throughput combination screening, we identified that the MEK inhibitor trametinib can be combined with BCL-2 family member inhibitors, to efficiently inhibit growth of neuroblastoma cell lines with RAS-MAPK mutations. Suppressing the RAS-MAPK pathway with trametinib led to an increase in pro-apoptotic BIM, resulting in more BIM binding to anti-apoptotic BCL-2 family members. By favoring the formation of these complexes, trametinib treatment enhances sensitivity to compounds targeting anti-apoptotic BCL-2 family members. *In vitro* validation studies confirmed that this sensitizing effect is dependent on an active RAS-MAPK pathway. *In vivo* combination of trametinib with BCL-2 inhibitors led to tumor inhibition in *NRAS*-mutant and *NF1*-deleted xenografts.

**Conclusion:**

Together, these results show that combining MEK inhibition with BCL-2 family member inhibition could potentially improve therapeutic outcomes for RAS-MAPK-mutated neuroblastoma patients.

## Introduction

Neuroblastoma is a pediatric tumor with a highly variable prognosis: low risk neuroblastoma tumors often regress without treatment, while high risk neuroblastoma tumors do not respond to intensive treatment regimens (1–4). Sequencing studies have revealed few recurrent events, and therefore little therapeutic opportunities (5–8). Neuroblastoma relapse tumors arise from primary tumors *via* clonal evolution and contain more aberrations than primary tumors (9, 10). These relapse tumors also showed an increase in targetable mutations, with the p53-MDM2-p14 ARF pathway, the *ALK* gene, and other RAS-MAPK pathway genes being frequently affected (10–13).

Previously, we have shown that neuroblastoma cell lines containing RAS-MAPK pathway mutations are sensitive to MEK inhibitors (10). However, when used *in vitro* these inhibitors reach a plateau phase where increasing the concentration does not further affect cell viability. *In vivo* this translates to only a slight tumor reduction. Furthermore, clinical trials have shown that MEK inhibitors as a monotherapy do not lead to sustainable responses (14, 15), warranting the search for an effective combination therapy.

Here, we identify through high-throughput drug screening that MEK inhibitors combined with BCL-2 family inhibitors induce apoptosis in RAS-MAPK-mutated neuroblastoma. *In vivo*, best results were obtained with the combination of trametinib and navitoclax in *RAS*-mutated SKNAS xenografts. Despite previously described limitations for navitoclax (16), observed partial responses (PRs) indicate that this is a combination therapy that should be further studied for the treatment of neuroblastoma tumors with aberrations in the RAS-MAPK pathway.

## Materials and methods

### Cell culture

Cell lines (derived from the American Type Culture Collection (ATCC)) were cultured in DMEM (Life Technologies, cat. 41965039) supplemented with 10% FBS (Life Technologies, cat. 10270106), 20 mM L-glutamine (Life Technologies, cat. 25030024), 10 U/ml penicillin and 10 μg/ml streptomycin (Life Technologies, cat. 15140122) and MEM Non-essential amino acids solution (100x, Life Technologies, Cat. No. 11140035), and maintained at 37°C under 5% CO_2_. Cell line identities were regularly confirmed by short tandem repeat (STR) profiling using the PowerPlex16 system and GeneMapper software (Promega) and cell lines were regularly screened for mycoplasma.

### High-throughput screens

Cells were seeded in 384-well plates (Corning, cat. 3764) at t=0, using the seeding densities shown in **Table 1**. At t=24 h trametinib or DMSO was added to a final concentration of 1 μM. Library compounds were added at final concentrations of 10 nM, 100 nM and 1 μM with appropriate solvent controls using the Sciclone ALH 3000 liquid handling robot. At t=96 h cell viability was determined using an MTT assay. The area under the curve was calculated using Graphpad Prism 6.

**Table 1:** Seeding densities in 384-well plates used for high-throughput drug screens.

Cell line	Number of cells per well
SJNB8	2000
KCNR	8000
SKNAS	2500
SKNBE	2000

The Sequoia anti-neoplastic library contains 157 drugs used in cancer treatment and was purchased from Sequoia Research Products. The SCREEN-WELL^®^ epigenetics library contains 43 epigenetic compounds and was acquired from Enzo Life Sciences. Other targeted inhibitors used in this study were purchased from Selleck Chemicals.

### Immunoprecipitation and western blotting

Samples were harvested in CHAPS buffer and subjected to co-immunoprecipitation (co-IP) using Dynabeads magnetic protein A agarose beads (Life Technologies) according to the manufacturer’s protocol. Immunoprecipitation antibodies were crosslinked to the beads using the Pierce BS3 crosslinker (ThermoFisher, cat. 21580). 1 mg of protein lysate was used as input material for co-IP reactions. Antibodies were acquired from Cell Signaling Technology unless noted otherwise. Antibodies were used for MEK (#8727), ERK (#9102), pMEK (#9154), pERK (#4376), BCL-2 (#4223), BCL-XL (#2764), MCL-1 (#4572), BIM (#2933), α-tubulin (#3873) and β-actin (Abcam, Ab6276).

### Cell viability assays

For cell viability assays, 5–20 × 10^3^ cells were seeded in 96-well plates, one day before treatment. Trametinib to a final concentration of 1 µM or DMSO was added to the cells and subsequently the BCL-2 family inhibitors were added in a seven-point fivefold dilution series. Cell viability was assayed by an MTT assay after 72 h (10). All experiments were performed in triplicate and values were compared to solvent-treated controls.

Bliss independence scores were calculated to measure synergy between trametinib and each of the tested concentrations of BCL-2 family inhibitors (17). Using the highest bliss score observed for each cell line, we subsequently calculated the average highest bliss score for the RAS-, NF1-, ALK, and non-mutated cell lines, respectively.

### *NRAS* overexpression construct

NMB cells expressing the doxycycline-inducible mutant *NRAS* Q61V were generated as previously described (10).

### Animal experiments

All experiments were conducted after obtaining ethical approval from the Dutch animal ethics committee of the Amsterdam UMC (SKNAS, DAG217) and the Netherlands Cancer Institute (KP-N-YN, AVD30100202011584, EGP10056). Single-cell suspensions containing 5 x 10^6^ cells of SKNAS or KP-N-YN neuroblastoma cell lines were generated in matrigel/DPBS (1:1) and transplanted subcutaneously into female NMRI nude mice (Charles River Laboratories (SKNAS), Janvier (KP-N-YN), 5-7 weeks old). Once the engrafted tumors reached a size of approximately 250 mm^3^, mice were randomly assigned to one of the following treatment arms (1): vehicle trametinib + vehicle venetoclax/navitoclax, (2) venetoclax + vehicle trametinib, (3) navitoclax + vehicle trametinib, (4) trametinib + vehicle venetoclax/navitoclax, (5) trametinib + venetoclax, and (6) trametinib + navitoclax. Trametinib (MedChem Express) was dissolved in 0.5% hydroxypropylmethylcellulose (Sigma) with 0.2% Tween-80 (Sigma) in MQ and dosed at 1 mg/kg/day *via* oral gavage. Venetoclax and navitoclax (MedChem Express) were dissolved in a vehicle consisting of 10% ethanol (Boom), 30% PEG400 (Sigma) and 60% Phosal (Lipoid). Both BCL-2 inhibitors were dosed at 100 mg/kg/day *via* oral gavage. Six to nine mice per treatment group received daily treatment during a period of 21 days or until humane endpoints were reached. In addition, two to three mice per group were treated for seven days and sacrificed 4 h after the last dose to harvest tumors for pharmacodynamic analysis. The number of mice per treatment arm is further specified in **Supplementary Table 1**. Tumor size was monitored two to three times a week, in a blinded manner. Tumor burden was determined according to the formula (π/6)d^3^, where d represents the mean tumor diameter obtained by caliper measurement. Statistical analysis was performed using linear mixed effects modeling followed by a pairwise analysis to compare the effects of single compound treatment to their respective combination therapies, with asterisks indicating a p-value <0.05.

## Results

### High throughput screening of RAS-MAPK-mutated cell lines in the presence and absence of trametinib

**Figure 1:**
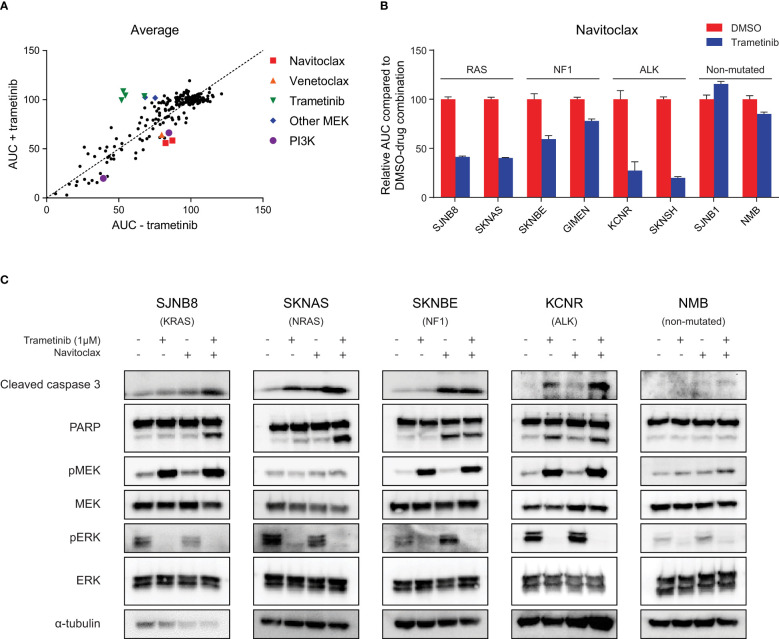
High throughput compound screening identifies navitoclax as a combination target with trametinib in neuroblastoma with RAS-MAPK mutations. **(A)** Relative area under the curve (AUC) for each screened compound in the presence of DMSO or trametinib averaged over four neuroblastoma cell lines (SJNB8, SKNAS, SKNBE, KCNR). **(B)** AUC for an extended panel of cell lines treated with seven concentrations of navitoclax combined with DMSO or trametinib. The AUC was normalized to the AUC of cells treated with navitoclax and DMSO. **(C)** Western blots of cell lines treated with DMSO, trametinib, navitoclax or the combination for 48 h.

Neuroblastoma cell lines that are treated with an increasing concentration of trametinib do not show complete cell death, but instead reach a plateau phase resulting from complete growth inhibition (**Supplementary Figure 1**). To identify compounds that are effective in combination with MEK inhibitors, we performed a large-scale drug screen. Four neuroblastoma cell lines were included based on the presence of the most common mutations affecting the RAS-MAPK pathway, *RAS* (*KRAS* in SJNB8 and *NRAS* in SKNAS), *NF1* (SKNBE) and *ALK* (KCNR). In total, 210 compounds were selected: 60 chemotherapeutic compounds, 106 targeted drugs that were either approved for clinical use or far along in (pre)clinical development, and 44 epigenetic modifiers (**Supplementary Table 2**). Cell lines were screened with 10 nM, 100 nM and 1 µM of the library compounds with and without 1 µM trametinib. Cell viability was normalized to solvent-treated cells in the DMSO assays and to the trametinib-treated cells in the trametinib assays. The area under the curve (AUC) for all compounds was determined in both assays and subsequently normalized to the AUC for DMSO-treated cells (**Supplementary Figure 2**). Most compounds were similarly effective in the DMSO- and trametinib-treated cells as evidenced by an average slope of 1.0 (range 0.95 tot 1.13) calculated by linear regression over all points per cell line (data not shown). As a negative control, we determined the effect of combining trametinib with other MEK inhibitors. In cells treated with trametinib, increased inhibition of RAS-MAPK signaling by the MEK inhibitors present in the library, showed no additional effect (**Figure 1A**, **Supplementary Figure 3**). Contrastingly, PI3K inhibitors are known to work synergistically with MEK inhibitors (18–20) and indeed had a larger effect when combined with trametinib. This shows that our methodology allows detection of synergistic and antagonistic effects.

### Trametinib treatment sensitizes RAS-MAPK-mutated neuroblastoma cells to navitoclax

To rank the compounds in their effectiveness, we calculated the average difference in AUC in the DMSO and trametinib assay over the four cell lines (**Figure 1A**, **Supplementary Table 3**). The most effective compound in combination with trametinib was navitoclax. Navitoclax is an inhibitor of the interaction between BIM and anti-apoptotic BCL-2 family members, showing strong affinity for BCL-2, BCL-xL and BCL-W (21). Its analog compound venetoclax, which specifically targets BCL-2 (22), showed a similar effect in the SKNAS and KCNR cell lines (**Supplementary Figure 3**). Interestingly, both navitoclax and venetoclax have previously shown effectivity in neuroblastoma cell lines and *in vivo* models (23–25). The combination with MEK inhibitors has also already been described as a synergistic combination in several other cancer types (26–28).

To confirm that this sensitizing effect also occurs in neuroblastoma cells with mutations in the RAS-MAPK pathway, we first evaluated the effect of trametinib in an extended panel of cell lines. Trametinib monotherapy inhibited cell growth more effectively in cell lines with RAS-MAPK mutations (**Supplementary Figure 4**). Exposing the RAS-MAPK-mutated cell lines to the combination of trametinib and navitoclax led to a clear sensitizing effect of trametinib on navitoclax sensitivity, while in non-mutated cell lines this was less pronounced (**Figure 1B**, **Supplementary Figure 5**). This suggests that the beneficial combined effect of MEK and BCL-2 family inhibition is dependent on an active RAS-MAPK pathway.

### Combination of trametinib with navitoclax enhances apoptosis in RAS-MAPK-mutated cell lines

MEK inhibition is expected to decrease phosphorylated ERK (pERK) and increase feedback loop-mediated upregulation of phosphorylated MEK (pMEK) (29, 30). This decrease in pERK was indeed observed upon treatment with trametinib, proving that the compound worked on-target in our set of neuroblastoma cell lines (**Figure 1C**). The expected increase in pMEK was also detected in most cell lines, except for NRAS-mutant SKNAS cells and wild-type NMB cells (**Figure 1C**). Despite the observed results, treating RAS-MAPK-mutated neuroblastoma cells with trametinib had mainly a cytostatic effect, as evidenced by the plateau phase at higher concentrations (**Supplementary Figure 1**). Combining trametinib with navitoclax is expected to enhance the apoptotic response. In line with this, largest increases in cleaved caspase 3 and cleaved PARP were generally observed upon combination of both compounds (**Figure 1C**). Next to testing these apoptotic markers, we also calculated the bliss independence scores as a measure for synergy. The average highest bliss score was 0.33 for the RAS-mutated lines, 0.15 for the NF1-mutated lines and 0.34 for the ALK-mutated lines. Cells without RAS-MAPK mutations, did not respond to the combination, as shown by the lack of cleaved caspase 3 and cleaved PARP in the NMB cells and an average highest bliss score of 0.08 for the wild-type cell lines (**Figure 1C**, **Supplementary Figure 5**). This further supports that the beneficial effect of combining trametinib and navitoclax is more pronounced in cells with overactivation of the RAS-MAPK pathway.

### Trametinib treatment induces upregulation of BIM

**Figure 2:**
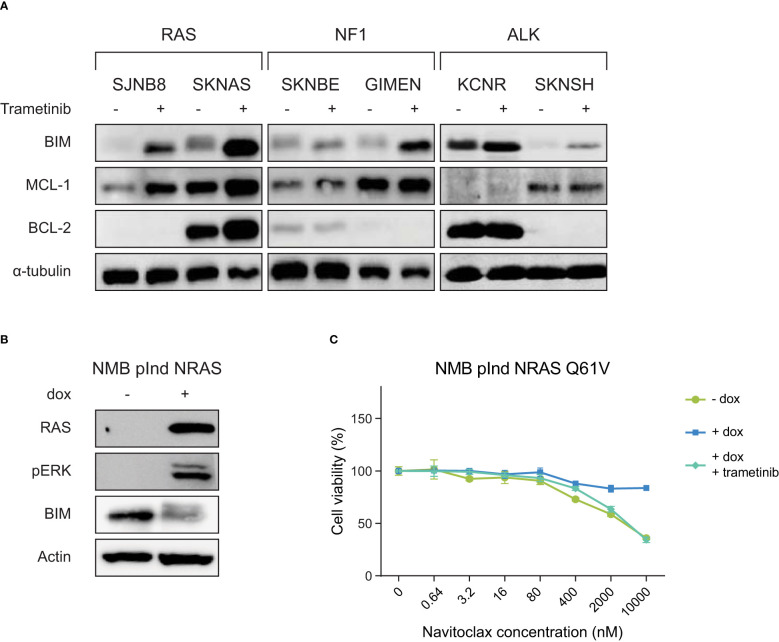
RAS-MAPK activation induces BIM destabilization and resistance to navitoclax, which can be inhibited by the MEK inhibitor trametinib. **(A)** Western blot of cell lines treated with DMSO or 1 µM trametinib for 6 h. Cell lines are grouped based on their mutation status of RAS-MAPK-associated genes. **(B)** Western blot of the inducible NRAS Q61V overexpression construct in the cell line NMB. **(C)** Navitoclax dose-response curves of NMB lines with inducible overexpression of NRAS Q61V with and without trametinib.

In cells with an active RAS-MAPK pathway, pERK is known to phosphorylate the pro-apoptotic protein BIM. Phosphorylated BIM (pBIM) is unstable and will be targeted for degradation in the proteasome, leading to an overall decrease in total BIM levels (**Supplementary Figure 6** (31)). To test whether this effect can be counteracted by MEK inhibition and could play a role in the synergy observed between trametinib and navitoclax, we studied how trametinib affects BIM levels. Trametinib treatment induced a clear increase in BIM protein levels in cell lines with mutations in *RAS*, *NF1* or *ALK* (**Figure 2A**). In line with **Figure 1C**, these results suggest that trametinib is capable of inhibiting pERK, thereby enhancing stabile non-phosphorylated BIM levels.

To validate whether the observed effect is dependent on an active RAS-MAPK pathway, we expressed a doxycycline-inducible mutant *NRAS Q61V* protein in the cell line NMB. NMB is a cell line without mutations in the RAS-MAPK pathway, causing this line to normally be insensitive to trametinib treatment. In addition, wild-type NMB cells show slight sensitivity to navitoclax treatment (**Figure 2C**, **Supplementary Figure 5**). Activation of the RAS-MAPK pathway by the addition of doxycycline, leads to a decrease in BIM levels (**Figure 2B**). As expected, this causes the *NRAS*-mutant NMB cells to become resistant to navitoclax treatment (**Figure 2C**). This effect could be abrogated by adding trametinib, indicating that the observed effects are indeed mediated by the RAS-MAPK pathway and not by other pathways downstream of RAS.

### Trametinib sensitizes neuroblastoma to various BCL-2 family member inhibitors

To further dissect whether trametinib would also sensitize cells towards other BH3 mimetics, we exposed our cell line panel to venetoclax, a specific BCL-2 inhibitor, and S63845, a specific MCL-1 inhibitor. In both cases, sensitivity was tested in the absence and presence of trametinib.

**Figure 3:**
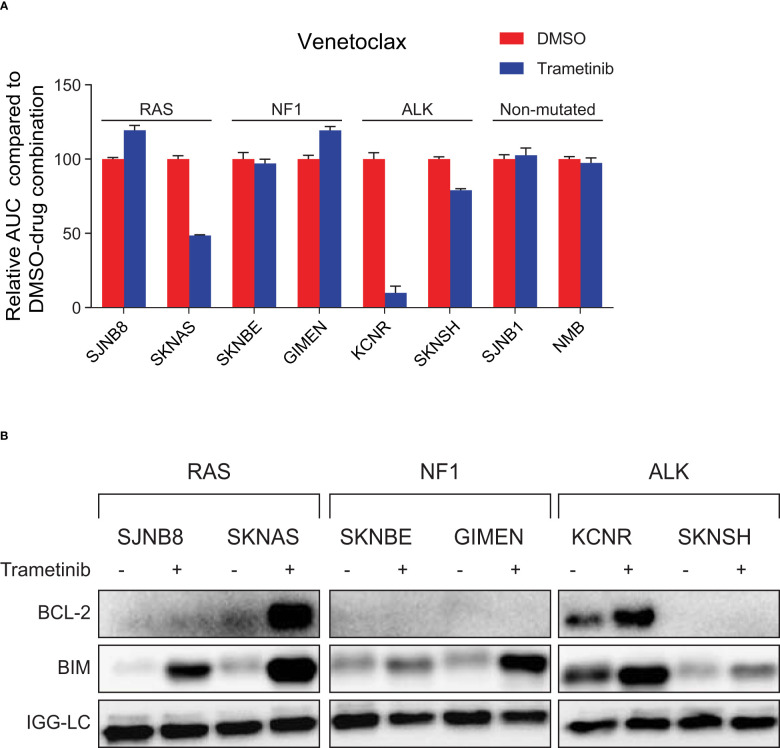
MEK inhibition sensitizes neuroblastoma cell lines with an active RAS-MAPK pathway and high BIM/BCL-2 complex expression to treatment with venetoclax. **(A)** Area under the curve (AUC) for a panel of cell lines treated with seven concentrations of the specific BCL-2 inhibitor venetoclax combined with DMSO or trametinib. The AUC is normalized to the AUC of cells treated with venetoclax and DMSO. **(B)** Co-immunoprecipitation of BIM-BCL-2 complexes in lines treated with DMSO or 1 µM trametinib for 6 h. Lysates were immunoprecipitated with a BIM antibody followed by western blotting for BCL-2.

Of the six cell lines with mutations in the RAS-MAPK pathway, SKNAS and KCNR were the only lines that were sensitized to venetoclax by trametinib treatment (**Figure 3A**, **Supplementary Figure 7**). Subsequent co-IP validation studies revealed that BIM is bound to BCL-2 in SKNAS and KCNR (**Figure 3B**), thereby explaining why synergistic effects were only observed in these lines. In KCNR, BIM is bound to BCL-2 irrespective of the presence of trametinib, while in SKNAS BIM is only bound to BCL-2 when trametinib is present.

Most RAS-MAPK-mutated lines were also sensitized to S63845 after trametinib treatment (**Supplementary Figure 8A**, **Supplementary Figure 9**). Co-IP of BIM in SJNB8 also shows an increase in MCL-1 bound to BIM when treated with trametinib (**Supplementary Figure 8B**). Overall, this suggests that increased BIM binding to anti-apoptotic proteins could be a general mechanism for the sensitizing effect of MEK inhibitors to various BH3 mimetics.

### Combining trametinib with BCL-2 family member inhibitors suppresses tumor growth *in vivo*

**Figure 4:**
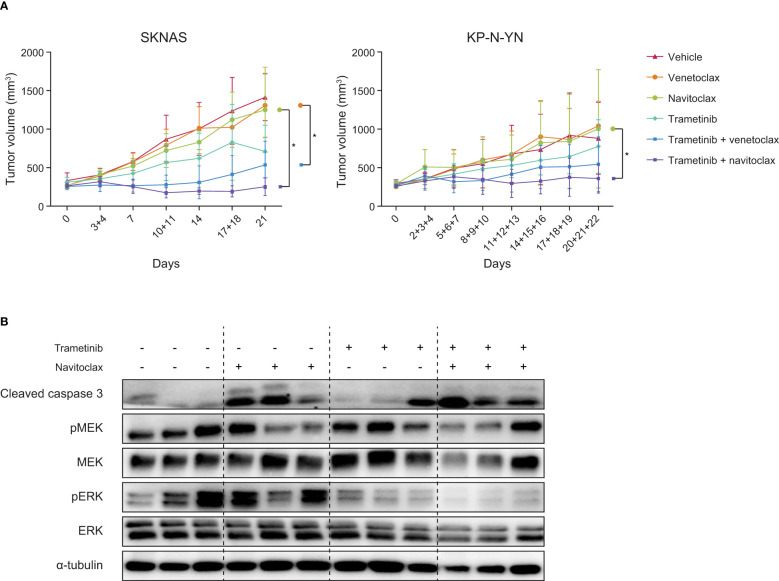
Combining navitoclax with trametinib enhances tumor growth inhibition in neuroblastoma xenografts. **(A)** Tumor growth curves of SKNAS and KP-N-YN xenografts treated with indicated compounds. Differences between treatment arms were analyzed using linear mixed effects modeling, with asterisks indicating a p-value <0.05. **(B)** Western blot of SKNAS xenografts treated for seven days with vehicle, navitoclax, trametinib or the combination. Tumors were harvested 4 h after the last dose.

Since navitoclax and venetoclax were both already in clinical development, we continued studying the effects of these two compounds in combination with trametinib. To validate the identified combinations *in vivo*, we first treated SKNAS xenografts with the combination of trametinib and navitoclax. The SKNAS model was expected to be sensitive to trametinib treatment due to the presence of an *NRAS Q61V* mutation. Over a treatment of 21 days, trametinib indeed appeared to cause tumor growth inhibition compared to vehicle-treated xenografts (**Figure 4A**), although significance was not reached (p=0.08). Navitoclax monotherapy had no clear effect on tumor growth in SKNAS xenografts, which was in line with our *in vitro* results (**Supplementary Figure 5**). Yet, combination with trametinib contributed to significant tumor regression (**Figure 4A**, **Supplementary Figure 10A**), with 6/6 mice showing PR as best response (32) (**Supplementary Table 4**). This indicates that the combination could hold potential for neuroblastoma tumors with *RAS* mutations.

Western blot analysis showed that the beneficial effect of the combination therapy can also be detected on a protein level. *In vivo* navitoclax treatment caused an increase in cleaved caspase 3, while trametinib decreased ERK phosphorylation (**Figure 4B**). Upon combination of navitoclax and trametinib, both effects were observed together. These responses are in line with our *in vitro* results, confirming that trametinib worked on-target and that the combination enhances apoptosis.

The same experimental set-up was used to test the combination of trametinib with the more specific BCL-2 inhibitor venetoclax. The combination induced tumor growth stabilization in the first stage of treatment, after which tumors slowly started growing again. Over time, trametinib-venetoclax combination-treated tumors were significantly smaller compared to tumors treated with venetoclax alone (**Figure 4A**), but best responses (2/6 PR, 3/6 SD, 1/6 PD (32)) were less powerful compared to tumors treated with the trametinib-navitoclax combination (**Supplementary Table 4**).

To further examine the applicability of these therapies in neuroblastoma with other RAS-MAPK mutations, both combinations were also tested in high BCL-2-expressing and *NF1*-mutant KP-N-YN cells. *In vitro* analysis of KP-N-YN showed a consistent response to trametinib monotherapy (**Supplementary Figure 11A**) and synergistic effects on cell viability after combination with either navitoclax (highest bliss score: 0.36) or venetoclax (highest bliss score: 0.40) (**Supplementary Figure 11B**). However, these *in vitro* results were not representative of the effects observed *in vivo*. Although *in vivo* trends were similar to the SKNAS model, results were less pronounced (trametinib + navitoclax: 4/9 PR, 3/9 SD, 2/9 PD and trametinib + venetoclax 3/8 PR, 2/8 SD, 3/8 PD) (**Figure 4A**, **Supplementary Figure 10B**, **Supplementary Table 4**). Significance was only reached between the navitoclax and trametinib-navitoclax treatment arms (**Figure 4A**).

## Discussion

We identified trametinib with navitoclax as a potential combination strategy for RAS-MAPK-mutated neuroblastoma. Trametinib sensitizes cells towards navitoclax by inhibiting pERK, thereby stabilizing and increasing BIM levels. This can be explained by a decrease in pERK-dependent phosphorylation and thereby less proteasomal degradation of BIM (31, 33). The increase in BIM is expected to benefit complex formation between BIM and anti-apoptotic BCL-2 family members, resulting in enhanced sensitivity towards navitoclax. Synergy between MEK inhibition and BH3 mimetics was previously reported in other tumor types (26, 34) where it was shown that *RAS* and *BRAF* mutations have destabilizing effects on BIM (35, 36). Here, we show that RAS-MAPK pathway activation through *RAS* mutation, *NF1* deletion or *ALK* mutation also has a destabilizing effect in neuroblastoma. This suggests that this is a common mechanism, that could be targeted by trametinib and navitoclax combination therapy, in a wide range of RAS-MAPK active cancers.

Navitoclax has shown great efficacy in preclinical studies (37, 38). However, in clinical trials it became apparent that inhibiting the binding between BIM and BCL-xL causes severe thrombocytopenia, limiting the therapeutic potential of this compound (16). An alternative is venetoclax, a drug that selectively inhibits binding of BCL-2 to BIM and therefore lacks the side-effects of navitoclax (22). Venetoclax has previously shown to be active in neuroblastoma cells with high BCL-2 expression and high BIM/BCL-2 complex levels (23, 39, 40). This is in line with the screen performed for this study, where venetoclax showed synergy with trametinib, but only in the cell lines that show a strong increase in BIM/BCL-2 complex levels after trametinib therapy. The combination of trametinib with venetoclax also led to a response *in vivo*. Although the effects were less pronounced compared to the combination with the more potent BCL-2 family inhibitor navitoclax, the lower toxic side-effects observed with venetoclax therapy might make this combination better suited for clinical use.

Another therapeutic option that should be further investigated, is a combination of trametinib with a low dose of navitoclax. This could potentially minimize the toxic side effects caused by navitoclax, while remaining the benefit of inhibiting BCL-2, BCL-xL and BCL-w instead of only BCL-2. Similar effects could also be obtained by adding trametinib to a lower dosed combination of both navitoclax and venetoclax. The combination of navitoclax and venetoclax is currently studied in pediatric patients with acute lymphocytic leukemia or lymphoblastic lymphoma (NCT05215405). Results of this study could give insight in whether a triple therapy with trametinib might be feasible.

We observed a heterogeneous response of our cell line panel to different targeted BCL-2 family inhibitors. Previously it was shown that sensitivity to these inhibitors is correlated to the anti-apoptotic binding partner of BIM (41). In primary neuroblastoma tumors a similar heterogeneity in BIM binding partners was observed (42), suggesting that not all tumors will respond to navitoclax or venetoclax and that different specific inhibitors should be used in conjunction with MEK inhibitors. For our *in vivo* studies, we decided to focus on the trametinib combinations with navitoclax and venetoclax, since these compounds were in clinical trial. Yet, our *in vitro* results suggest that the MCL-1 inhibitor S68345 could also be effective in a subset of neuroblastoma tumors with lower BCL-2 levels, where BIM is bound to MCL-1. At the moment, several other MCL-1 inhibitors (e.g. MIK665, AZD5991 and AMG176) are tested in clinical trials. Therefore, it would be interesting to further investigate the effectivity of combined MEK and MCL-1 inhibition in neuroblastoma *in vivo* models.

Our study also indicates that the therapeutic efficacy of the combination could vary based on the type of RAS-MAPK mutation present. *RAS*-mutated SKNAS xenografts were more sensitive to the combination therapy than *NF1*-mutated KP-N-YN tumors. These results were in line with previous *in vitro* studies (10), and our own data, comparing the SJNB8 and SKNAS lines (*RAS* mutants) to SKNBE and GIMEN (*NF1* mutants). However, it must be noted that *in vitro* results of the *NF1*-mutated cell line KP-N-YN showed higher bliss scores than the other *NF1*-mutated lines that we tested. In fact, the highest bliss score of KP-N-YN was similar to the *RAS-*mutated lines. Hence, the synergistic *in vitro* effects of KP-N-YN do not match with the observed *in vivo* responses. This indicates that there are more factors playing a role in the sensitivity differences of model systems. For follow-up studies, it would for instance be interesting to also examine *in vivo* BIM-BCL-2 complex levels, since this could potentially explain efficacy differences between our xenograft models. It also underscores the need to further investigate the effects on different subgroups of RAS-MAPK-mutated tumors, in a larger panel of cell lines and *in vivo* models.

Relapse neuroblastoma tumors frequently show RAS-MAPK mutations, and in primary tumors this pathway is commonly activated as well (6, 10, 43). Since most neuroblastoma tumors also demonstrate high levels of BCL-2 (23, 44), a significant part of neuroblastoma patients could benefit from the combination of MEK inhibitors and BCL-2 inhibitors. Both trametinib (NCT02124772) and venetoclax (NCT03236857) are separately studied in phase I clinical trials for neuroblastoma. Combinations of trametinib and navitoclax are currently also studied in phase I/II trials (NCT02079740 and NCT01989585) for other cancer types in adults. These studies will clarify whether the compounds are tolerable and safe to use, and some of them will hopefully give insight in whether the compounds are effective in RAS-MAPK-mutated and high BCL-2-expressing tumors, respectively. In case both compounds are shown to work on-target, we would propose to combine trametinib with navitoclax and/or venetoclax in a clinical trial, since our study suggests that this could increase compound efficacy in a major subgroup of neuroblastoma.

## Data availability statement

The original contributions presented in the study are included in the article/**Supplementary material**. Further inquiries can be directed to the corresponding author.

## Ethics statement

The animal studies were reviewed and approved by the Dutch animal ethics committee of the Amsterdam UMC and the Netherlands Cancer Institute.

## Author contributions

Conceptualization: TE, LV, RV, ED, JM. Investigation: TE, LV, LS, BK, LA, ME, ED. Validation: TE, LV, LS, RD. Supervision: HT, HC, MN, GT, SE, RV, ED, JM. Writing – original draft: TE, LV. Writing – review and editing: HC, JK, MN, GT, SE, RV, ED, JM. Resources: JK. Funding acquisition: ED, JM. All authors contributed to the article and approved the submitted version.

## References

[1] Newman EA Abdessalam S Aldrink JH Austin M Heaton TE Bruny J. Update on neuroblastoma. J Pediatr Surg (2019) 54(3):383–9. doi: 10.1016/j.jpedsurg.2018.09.004 30305231

[2] Sokol E Desai AV. The evolution of risk classification for neuroblastoma. Children (Basel) (2019) 6(2):27. doi: 10.3390/children6020027 30754710 PMC6406722

[3] Tas ML Nagtegaal M Kraal K Tytgat GAM Abeling N Koster J. Neuroblastoma stage 4s: Tumor regression rate and risk factors of progressive disease. Pediatr Blood Cancer (2020) 67(4):e28061. doi: 10.1002/pbc.28061 31736229

[4] Ackermann S Cartolano M Hero B Welte A Kahlert Y Roderwieser A. A mechanistic classification of clinical phenotypes in neuroblastoma. Science (2018) 362(6419):1165–70. doi: 10.1126/science.aat6768 PMC7875194 30523111

[5] Molenaar JJ Koster J Zwijnenburg DA van Sluis P Valentijn LJ van der Ploeg I. Sequencing of neuroblastoma identifies chromothripsis and defects in neuritogenesis genes. Nature (2012) 483(7391):589–93. doi: 10.1038/nature10910 22367537

[6] Pugh TJ Morozova O Attiyeh EF Asgharzadeh S Wei JS Auclair D. The genetic landscape of high-risk neuroblastoma. Nat Genet (2013) 45(3):279–84. doi: 10.1038/ng.2529 PMC3682833 23334666

[7] Cheung N-KV Zhang J Lu C Parker M Bahrami A Tickoo SK. Association of age at diagnosis and genetic mutations in patients with neuroblastoma. Jama (2012) 307(10):1062–71. doi: 10.1001/jama.2012.228 PMC3527076 22416102

[8] Sausen M Leary RJ Jones S Wu J Reynolds CP Liu X. Integrated genomic analyses identify Arid1a and Arid1b alterations in the childhood cancer neuroblastoma. Nat Genet (2013) 45(1):12–7. doi: 10.1038/ng.2493 PMC3557959 23202128

[9] Schramm A Koster J Assenov Y Althoff K Peifer M Mahlow E. Mutational dynamics between primary and relapse neuroblastomas. Nat Genet (2015) 47(8):872–7. doi: 10.1038/ng.3349 26121086

[10] Eleveld TF Oldridge DA Bernard V Koster J Daage LC Diskin SJ. Relapsed neuroblastomas show frequent ras-mapk pathway mutations. Nat Genet (2015) 47(8):864–71. doi: 10.1038/ng.3333 PMC4775079 26121087

[11] Carr-Wilkinson J O'Toole K Wood KM Challen CC Baker AG Board JR. High frequency of P53/Mdm2/P14^ARF^ pathway abnormalities in relapsed neuroblastoma. Clin Cancer Res (2010) 16(4):1108–18. doi: 10.1158/1078-0432.ccr-09-1865 PMC2842933 20145180

[12] Padovan-Merhar OM Raman P Ostrovnaya I Kalletla K Rubnitz KR Sanford EM. Enrichment of targetable mutations in the relapsed neuroblastoma genome. PloS Genet (2016) 12(12):e1006501. doi: 10.1371/journal.pgen.1006501 27997549 PMC5172533

[13] Schleiermacher G Javanmardi N Bernard V Leroy Q Cappo J Rio Frio T. Emergence of new alk mutations at relapse of neuroblastoma. J Clin Oncol (2014) 32(25):2727–34. doi: 10.1200/JCO.2013.54.0674 25071110

[14] Flaherty KT Robert C Hersey P Nathan P Garbe C Milhem M. Improved survival with mek inhibition in braf-mutated melanoma. New Engl J Med (2012) 367(2):107–14. doi: 10.1056/NEJMoa1203421 22663011

[15] Kim KB Kefford R Pavlick AC Infante JR Ribas A Sosman JA. Phase ii study of the Mek1/Mek2 inhibitor trametinib in patients with metastatic braf-mutant cutaneous melanoma previously treated with or without a braf inhibitor. J Clin Oncol (2012) 31(4):482–9. doi: 10.1200/JCO.2012.43.5966 PMC4878037 23248257

[16] Wilson WH O'Connor OA Czuczman MS LaCasce AS Gerecitano JF Leonard JP. Navitoclax, a targeted high-affinity inhibitor of bcl-2, in lymphoid malignancies: A phase 1 dose-escalation study of safety, pharmacokinetics, pharmacodynamics, and antitumour activity. Lancet Oncol (2010) 11(12):1149–59. doi: 10.1016/S1470-2045(10)70261-8 PMC3025495 21094089

[17] Zhao W Sachsenmeier K Zhang L Sult E Hollingsworth RE Yang H. A new bliss independence model to analyze drug combination data. J Biomol Screen (2014) 19(5):817–21. doi: 10.1177/1087057114521867 24492921

[18] Haagensen EJ Kyle S Beale GS Maxwell RJ Newell DR. The synergistic interaction of mek and Pi3k inhibitors is modulated by mtor inhibition. Br J Cancer (2012) 106(8):1386–94. doi: 10.1038/bjc.2012.70 PMC3326670 22415236

[19] Vujic I Posch C Sanlorenzo M Yen AJ Tsumura A Kwong A. Mutant Nrasq61 shares signaling similarities across various cancer types–potential implications for future therapies. Oncotarget (2014) 5(17):7936–44. doi: 10.18632/oncotarget.2326 PMC4202171 25277205

[20] Chang R Tosi U Voronina J Adeuyan O Wu LY Schweitzer ME. Combined targeting of Pi3k and mek effector pathways *Via* ced for dipg therapy. Neurooncol Adv (2019) 1(1):vdz004. doi: 10.1093/noajnl/vdz004 32642647 PMC7212917

[21] Tse C Shoemaker AR Adickes J Anderson MG Chen J Jin S. Abt-263: A potent and orally bioavailable bcl-2 family inhibitor. Cancer Res (2008) 68(9):3421–8. doi: 10.1158/0008-5472.can-07-5836 18451170

[22] Souers AJ Leverson JD Boghaert ER Ackler SL Catron ND Chen J. Abt-199, a potent and selective bcl-2 inhibitor, achieves antitumor activity while sparing platelets. Nat Med (2013) 19(2):202–8. doi: 10.1038/nm.3048 23291630

[23] Bate-Eya LT den Hartog IJM van der Ploeg I Schild L Koster J Santo EE. High efficacy of the bcl-2 inhibitor Abt199 (Venetoclax) in bcl-2 high-expressing neuroblastoma cell lines and xenografts and rational for combination with mcl-1 inhibition. Oncotarget (2016) 7(19):27946–58. doi: 10.18632/oncotarget.8547 PMC5053701 27056887

[24] Ham J Costa C Sano R Lochmann TL Sennott Erin M Patel Neha U. Exploitation of the apoptosis-primed state of mycn-amplified neuroblastoma to develop a potent and specific targeted therapy combination. Cancer Cell (2016) 29(2):159–72. doi: 10.1016/j.ccell.2016.01.002 PMC4749542 26859456

[25] Lamers F Schild L den Hartog IJM Ebus ME Westerhout EM Ora I. Targeted Bcl2 inhibition effectively inhibits neuroblastoma tumour growth. Eur J Cancer (2012) 48(16):3093–103. doi: 10.1016/j.ejca.2012.01.037 22366560

[26] Tan N Wong M Nannini MA Hong R Lee LB Price S. Bcl-2/Bcl-Xl inhibition increases the efficacy of mek inhibition alone and in combination with Pi3 kinase inhibition in lung and pancreatic tumor models. Mol Cancer Ther (2013) 12(6):853–64. doi: 10.1158/1535-7163.MCT-12-0949 23475955

[27] Corcoran RB Cheng KA Hata AN Faber AC Ebi H Coffee EM. Synthetic lethal interaction of combined bcl-xl and mek inhibition promotes tumor regressions in kras mutant cancer models. Cancer Cell (2013) 23(1):121–8. doi: 10.1016/j.ccr.2012.11.007 PMC3667614 23245996

[28] Han L Zhang Q Shi C Cavazos A Ruvolo V Leverson J. Targeting mapk signaling pathway with cobimetinib (Gdc-0973) enhances anti-leukemia efficacy of venetoclax (Abt-199/Gdc-0199) in acute myeloid leukemia models. Blood (2016) 128(22):97. doi: 10.1182/blood.V128.22.97.97

[29] Hatzivassiliou G Haling JR Chen H Song K Price S Heald R. Mechanism of mek inhibition determines efficacy in mutant kras- versus braf-driven cancers. Nature (2013) 501(7466):232–6. doi: 10.1038/nature12441 23934108

[30] Jin XF Spottl G Maurer J Nolting S Auernhammer CJ. Antitumoral activity of the mek inhibitor trametinib (Tmt212) alone and in combination with the Cdk4/6 inhibitor ribociclib (Lee011) in neuroendocrine tumor cells in vitro. Cancers (Basel) (2021) 13(6):1485. doi: 10.3390/cancers13061485 33807122 PMC8004919

[31] Ley R Ewings KE Hadfield K Cook SJ. Regulatory phosphorylation of bim: Sorting out the erk from the jnk. Cell Death Differ (2005) 12(8):1008–14. doi: 10.1038/sj.cdd.4401688 15947788

[32] Eisenhauer EA Therasse P Bogaerts J Schwartz LH Sargent D Ford R. New response evaluation criteria in solid tumours: Revised recist guideline (Version 1.1). Eur J Cancer (2009) 45(2):228–47. doi: 10.1016/j.ejca.2008.10.026 19097774

[33] Alcon C Martin F Prada E Mora J Soriano A Guillen G. Mek and mcl-1 sequential inhibition synergize to enhance rhabdomyosarcoma treatment. Cell Death Discovery (2022) 8(1):172. doi: 10.1038/s41420-022-00959-w 35393436 PMC8989976

[34] Cragg MS Jansen ES Cook M Harris C Strasser A Scott CL. Treatment of b-raf mutant human tumor cells with a mek inhibitor requires bim and is enhanced by a Bh3 mimetic. J Clin Invest (2008) 118(11):3651–9. doi: 10.1172/jci35437 PMC2571034 18949058

[35] Tan T-T Degenhardt K Nelson DA Beaudoin B Nieves-Neira W Bouillet P. Key roles of bim-driven apoptosis in epithelial tumors and rational chemotherapy. Cancer Cell (2005) 7(3):227–38. doi: 10.1016/j.ccr.2005.02.008 15766661

[36] Wickenden JA Jin H Johnson M Gillings AS Newson C Austin M. Colorectal cancer cells with the Brafv600e mutation are addicted to the Erk1/2 pathway for growth factor-independent survival and repression of bim. Oncogene (2008) 27(57):7150–61. doi: 10.1038/onc.2008.335 PMC2643813 18806830

[37] Suryani S Carol H Chonghaile TN Frismantas V Sarmah C High L. Cell and molecular determinants of *In vivo* efficacy of the Bh3 mimetic abt-263 against pediatric acute lymphoblastic leukemia xenografts. Clin Cancer Res (2014) 20(17):4520–31. doi: 10.1158/1078-0432.ccr-14-0259 PMC4154988 25013123

[38] Lock R Carol H Houghton PJ Morton CL Kolb EA Gorlick R. Initial testing (Stage 1) of the Bh3 mimetic abt-263 by the pediatric preclinical testing program. Pediatr Blood Cancer (2008) 50(6):1181–9. doi: 10.1002/pbc.21433 18085673

[39] Vernooij L Bate-Eya LT Alles LK Lee JY Koopmans B Jonus HC. High-throughput screening identifies idasanutlin as a resensitizing drug for venetoclax-resistant neuroblastoma cells. Mol Cancer Ther (2021) 20(6):1161–72. doi: 10.1158/1535-7163.MCT-20-0666 PMC7611269 33850004

[40] Tanos R Karmali D Nalluri S Goldsmith KC. Select bcl-2 antagonism restores chemotherapy sensitivity in high-risk neuroblastoma. BMC Cancer (2016) 16:97. doi: 10.1186/s12885-016-2129-0 26874859 PMC4752777

[41] Goldsmith KC Lestini BJ Gross M Ip L Bhumbla A Zhang X. Bh3 response profiles from neuroblastoma mitochondria predict activity of small molecule bcl-2 family antagonists. Cell Death different (2010) 17(5):872–82. doi: 10.1038/cdd.2009.171 PMC3690273 19893570

[42] Goldsmith KC Gross M Peirce S Luyindula D Liu X Vu A. Mitochondrial bcl-2 family dynamics define therapy response and resistance in neuroblastoma. Cancer Res (2012) 72(10):2565–77. doi: 10.1158/0008-5472.CAN-11-3603 PMC3354953 22589275

[43] Fransson S Martinez-Monleon A Johansson M Sjoberg RM Bjorklund C Ljungman G. Whole-genome sequencing of recurrent neuroblastoma reveals somatic mutations that affect key players in cancer progression and telomere maintenance. Sci Rep (2020) 10(1):22432. doi: 10.1038/s41598-020-78370-7 33384420 PMC7775426

[44] Bierbrauer A Jacob M Vogler M Fulda S. A direct comparison of selective Bh3-mimetics reveals bcl-xl, bcl-2 and mcl-1 as promising therapeutic targets in neuroblastoma. Br J Cancer (2020) 122(10):1544–51. doi: 10.1038/s41416-020-0795-9 PMC7217842 32203216

