# Supplementary material for: MEK inhibition causes BIM stabilization and increased sensitivity to BCL-2 family member inhibitors in RAS-MAPK-mutated neuroblastoma
Source: Front Oncol. 2023 Feb 21;13:1130034. doi: 10.3389/fonc.2023.1130034 (PMC9990464; doi:10.3389/fonc.2023.1130034)
Supplement: Supplementary file 1 [file DataSheet_1.docx]

Supplementary Material


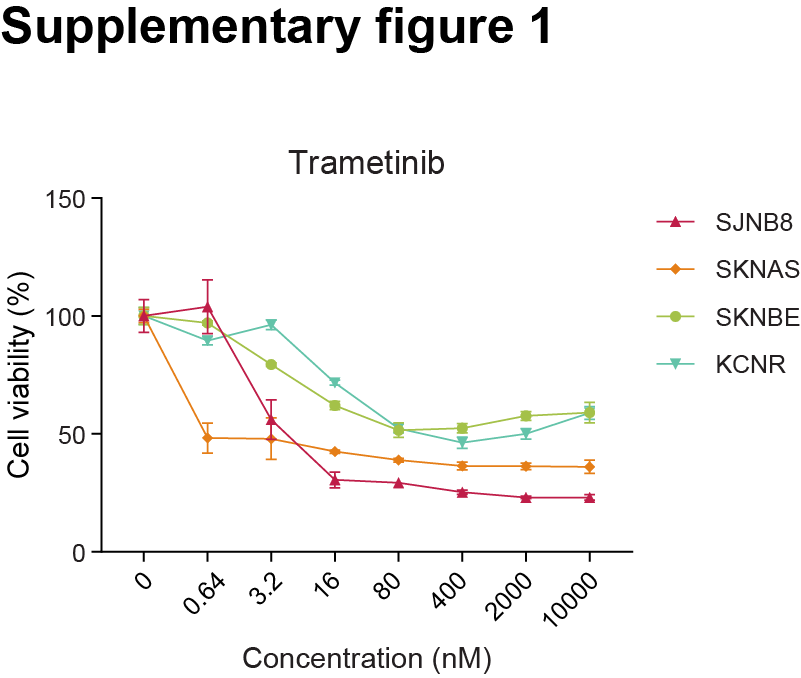


**Supplementary Figure 1:** Cell viability curves of cell lines used in the drug screen treated with a

concentration range of trametinib.


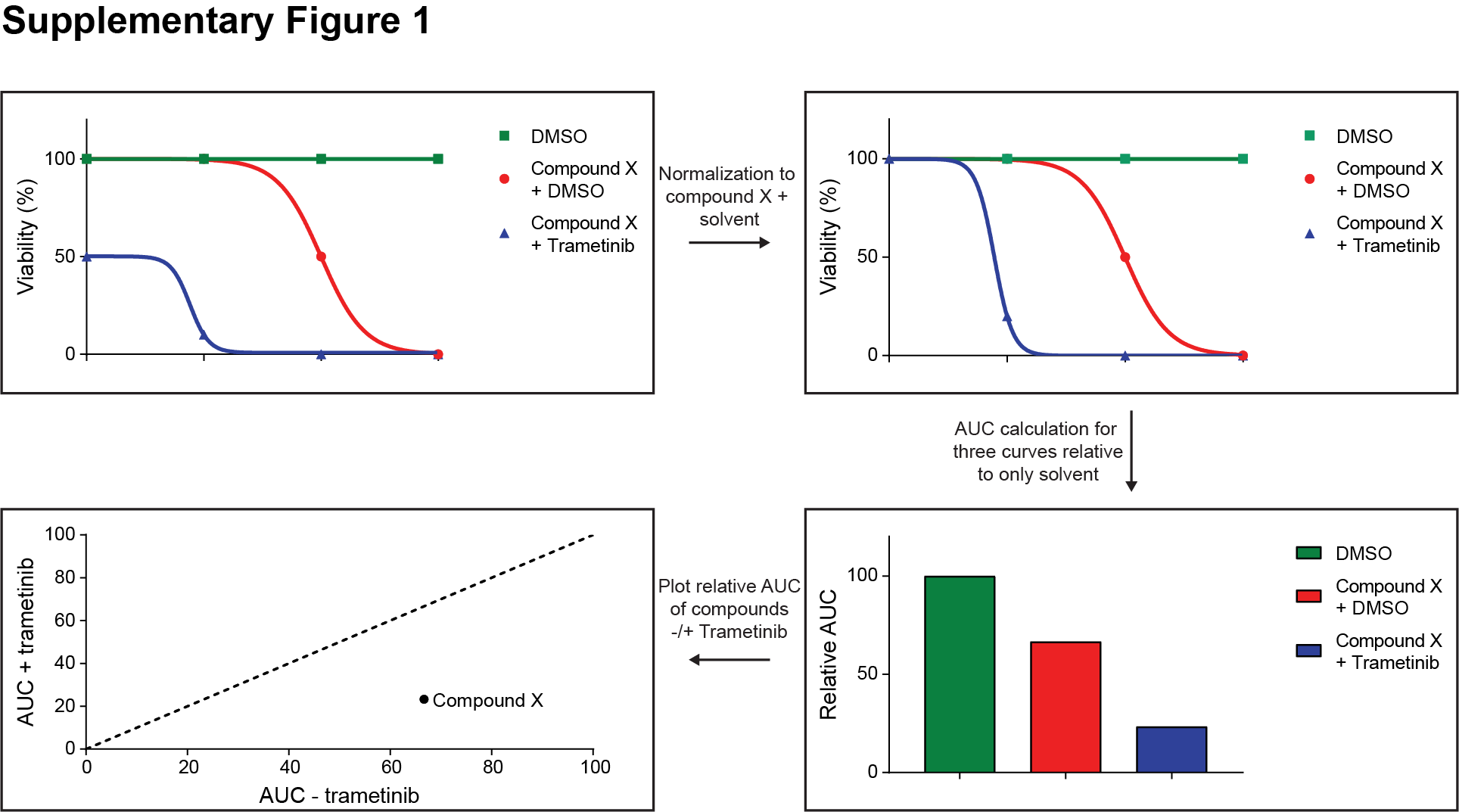


**Supplementary Figure 2:** Methodology for calculating the relative area under the curve from viability curves.


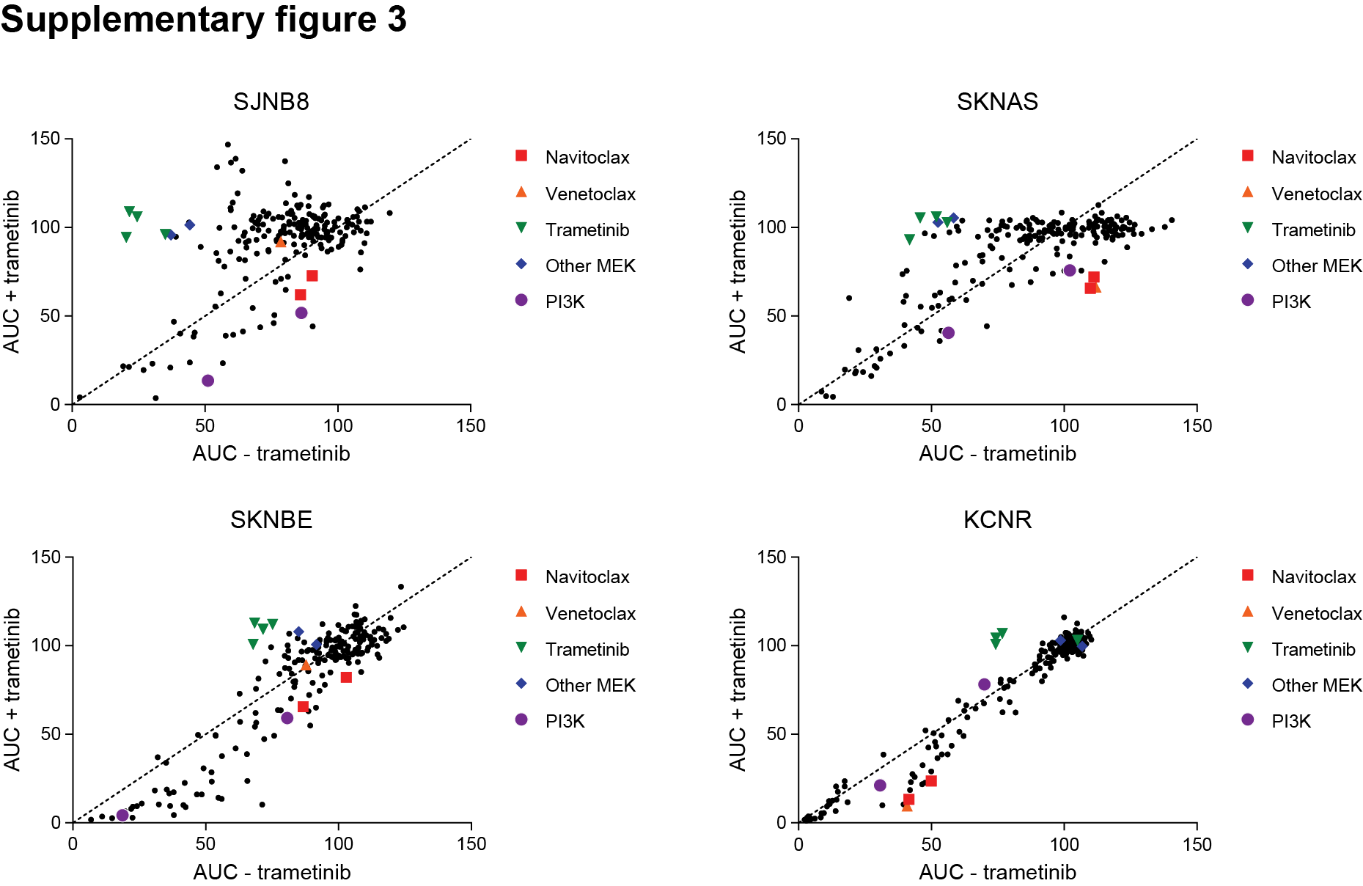


**Supplementary Figure 3:** Relative area under the curve for each screened compound in the presence of DMSO or trametinib in four neuroblastoma cell lines. Values were calculated as shown in Supplementary Figure 2.


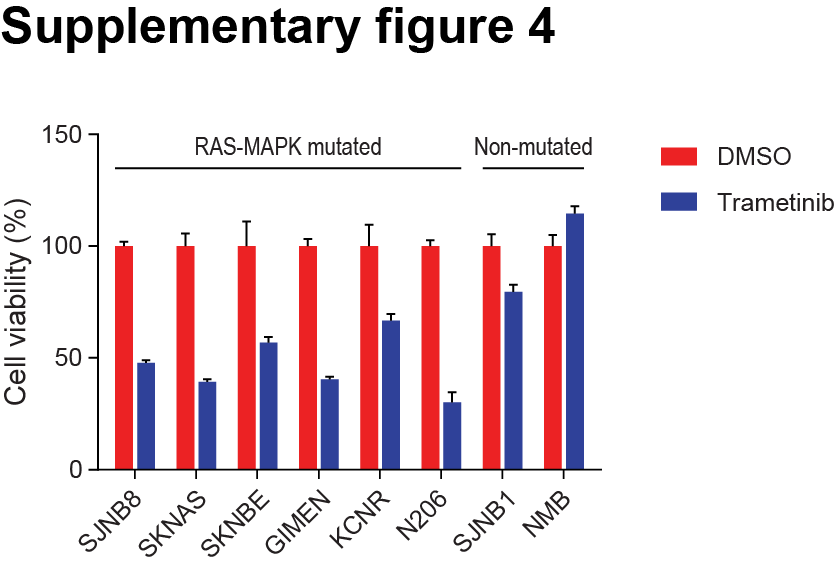


**Supplementary Figure 4:** Viability of extended cell line panel treated with 1 µM of trametinib or equivalent amounts of DMSO. Both conditions were normalized to DMSO-treated cells.


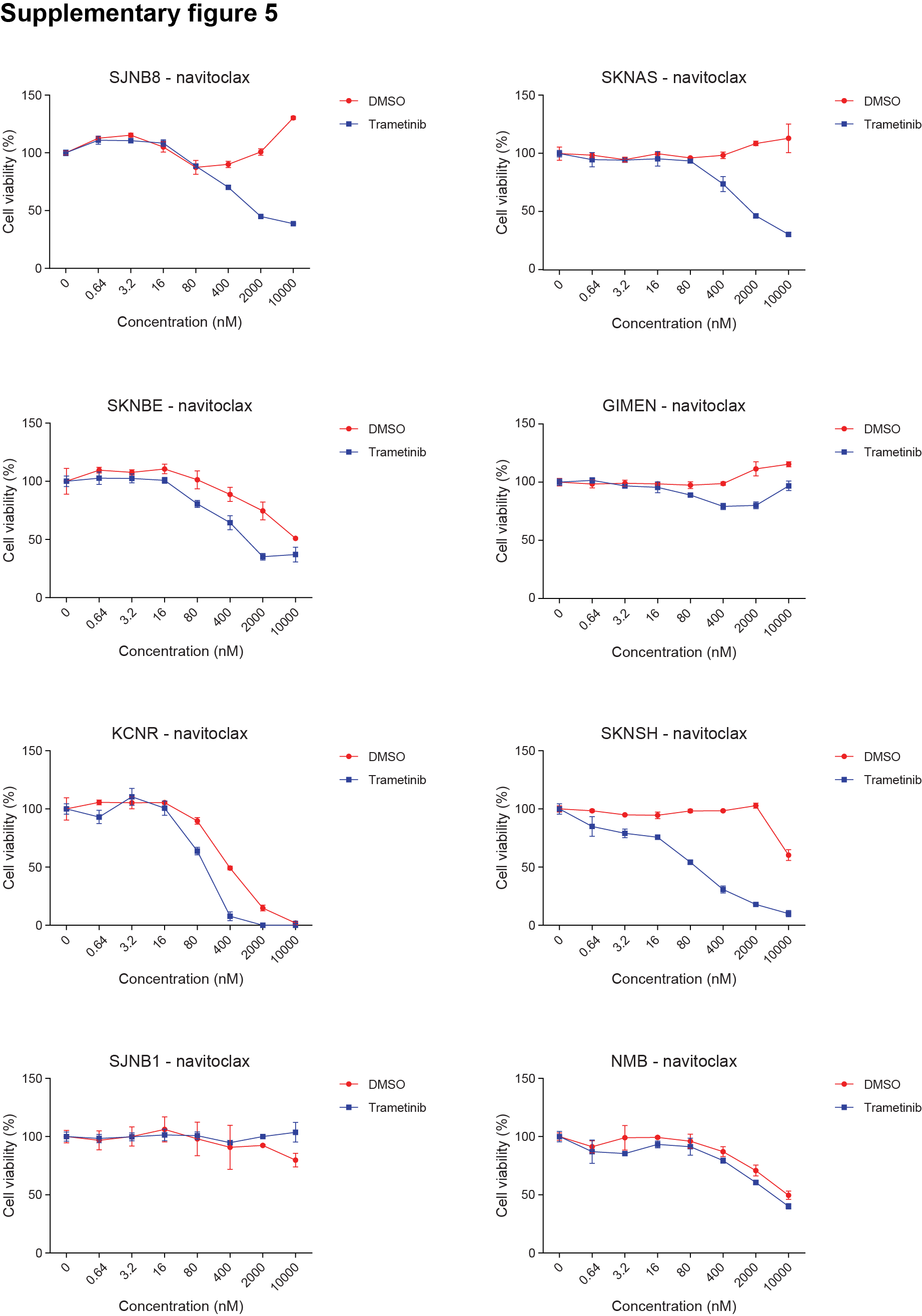


**Supplementary Figure 5:** Cell viability curves for navitoclax in the presence and absence of 1 µM trametinib. Values were normalized to trametinib- and DMSO-treated cells, respectively.


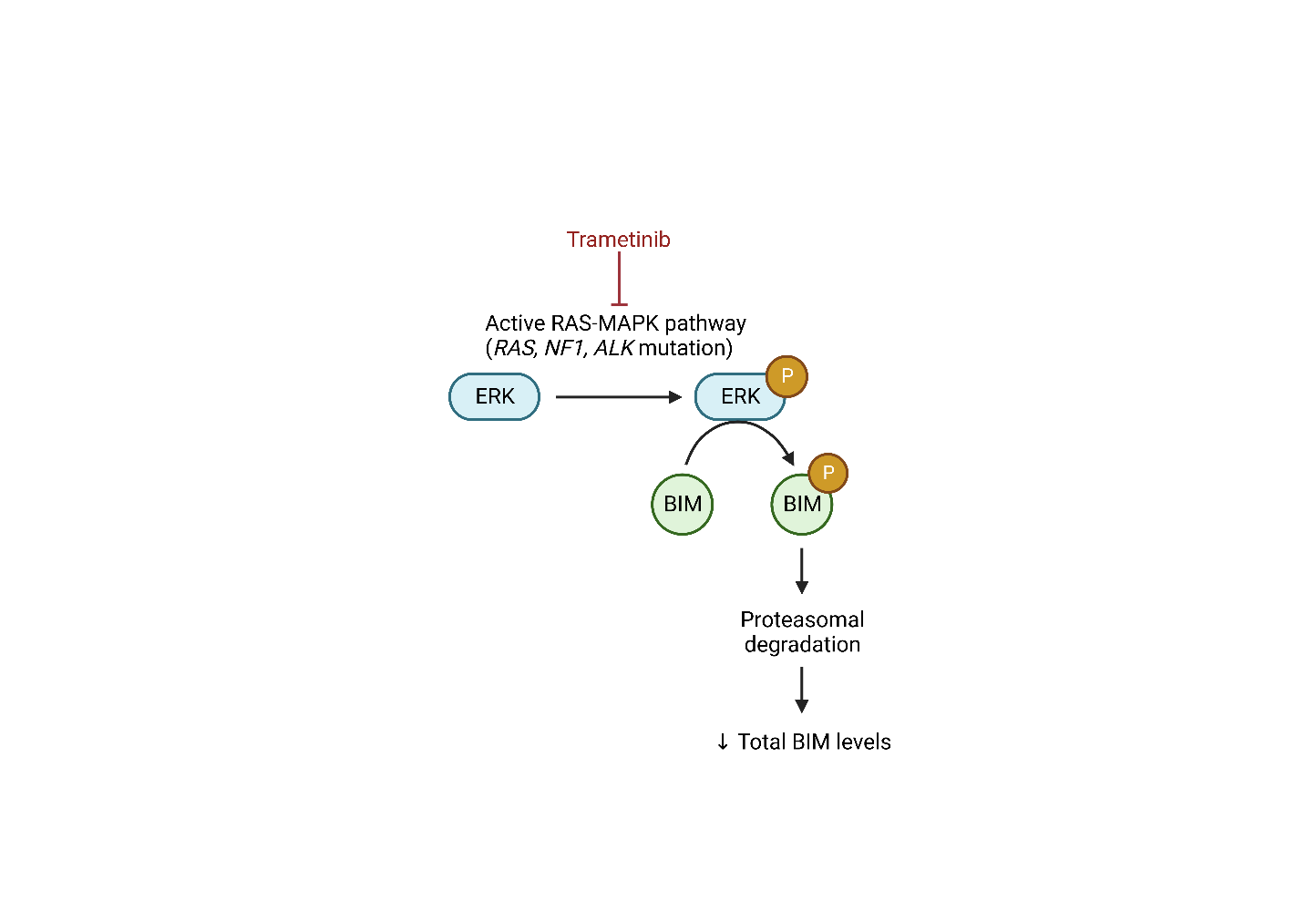


**Supplementary Figure 6:** Mechanistic overview of trametinib-induced increase in BIM. This figure is created with BioRender.com and based on information provided by [31].


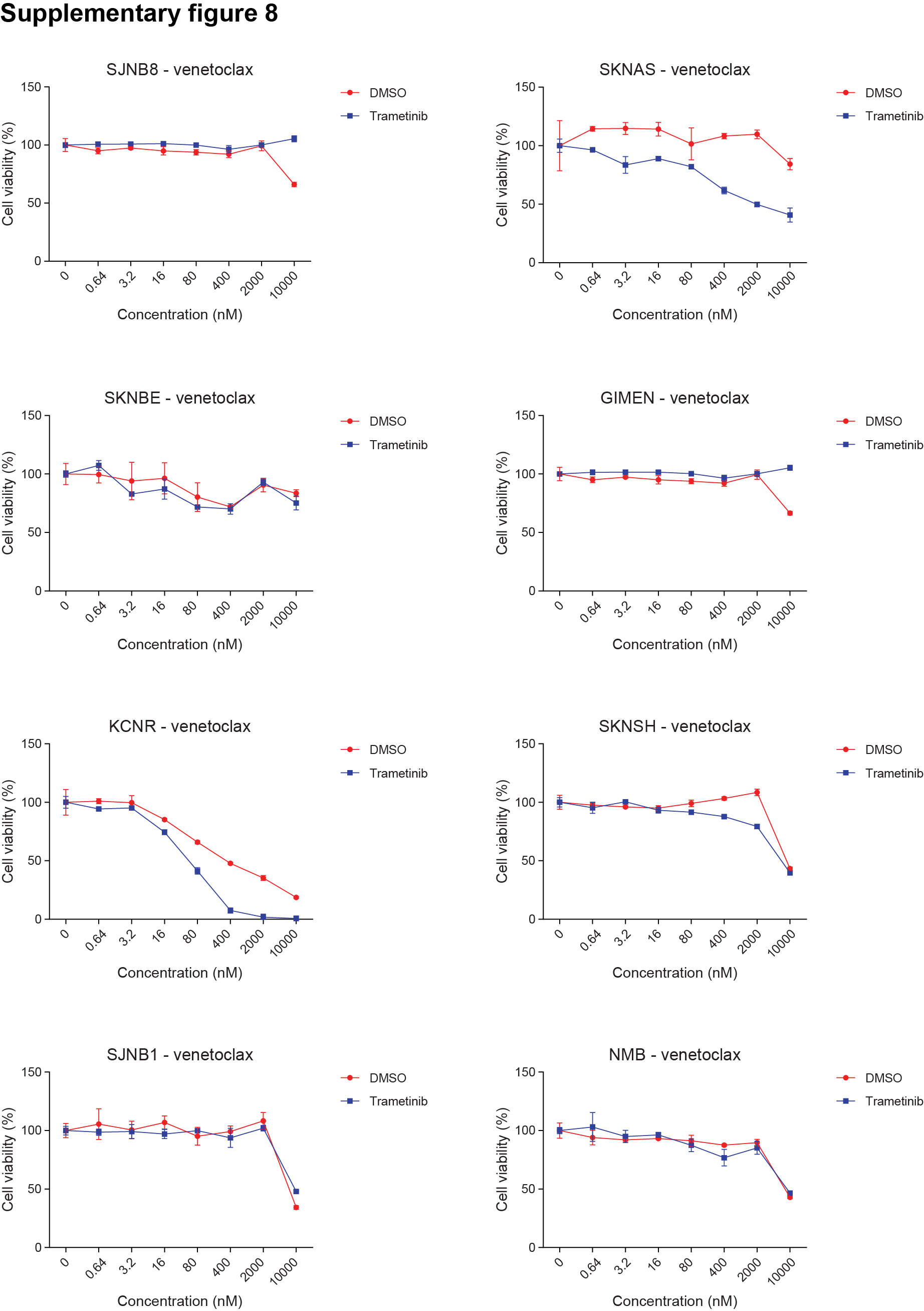


**Supplementary Figure 7:** Cell viability curves for venetoclax in the presence and absence of 1 µM trametinib. Values were normalized to trametinib- and DMSO-treated cells, respectively.


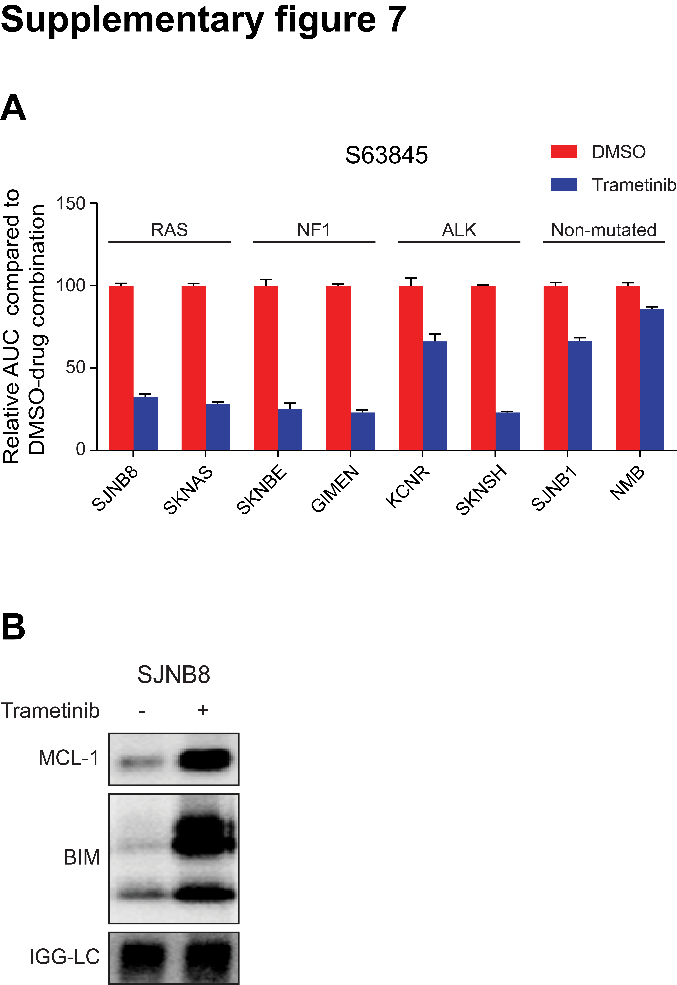


**Supplementary Figure 8:** MEK inhibition sensitizes neuroblastoma cell lines with an active RAS-MAPK pathway to treatment with the MCL-1 inhibitor S63845. **(A)** Area under the curve (AUC) for a panel of cell lines treated with seven concentrations of the MCL-1 inhibitor S63845 combined with either DMSO or trametinib. The AUC was normalized to the AUC of cells treated with S63845 and DMSO. **(B)** MCL-1/BIM complex levels in SJNB8 cells treated with trametinib or equivalent amounts of DMSO. Complex levels were obtained by immunoprecipitation of BIM followed by western blotting for MCL-1.
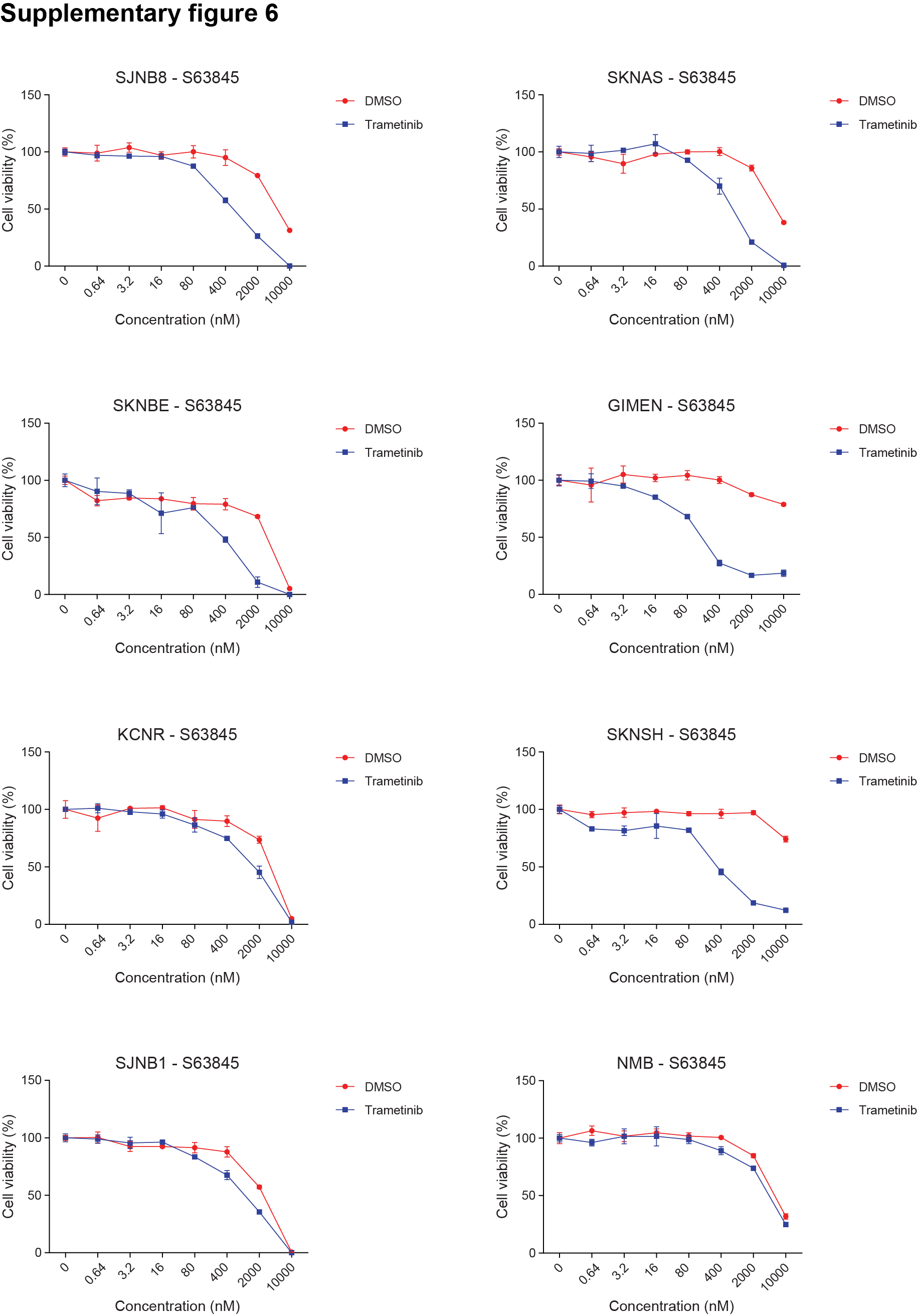


**Supplementary Figure 9:** Cell viability curves for S63845 in the presence and absence of 1 µM trametinib. Values were normalized to trametinib- and DMSO-treated cells, respectively.


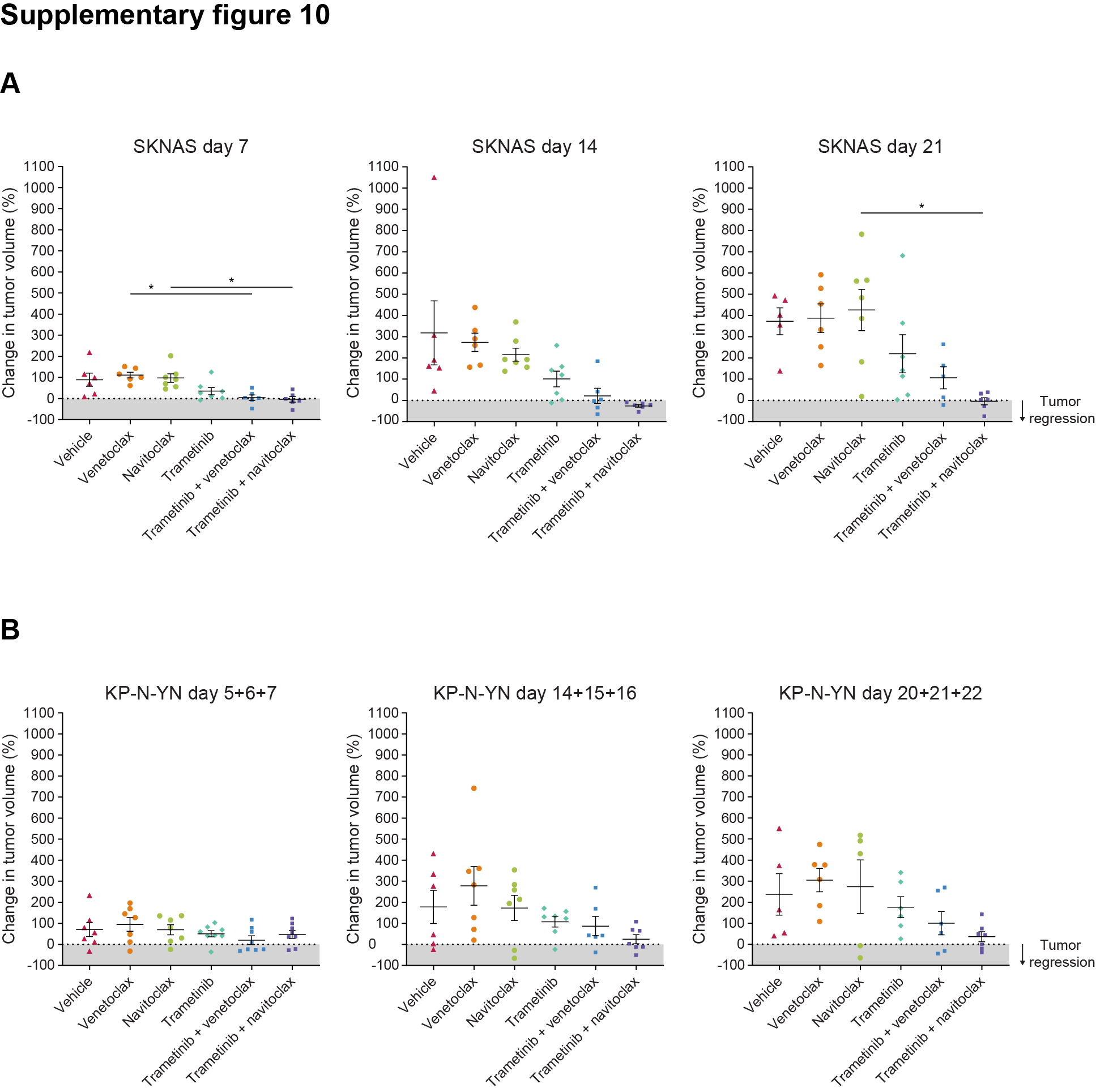


**Supplementary Figure 10:** *In vivo* analysis of the combinations of trametinib with navitoclax or venetoclax. **(A)** Percentage change in tumor volume compared to the start of treatment in SKNAS xenografts at day 7, 14 and 21 of treatment. **(B)** Percentage change in tumor volume compared to the start of treatment in KP-N-YN xenografts at day 7, 14 and 21 of treatment. Asterisks indicate a p-value <0.05 calculated by ANOVA with Tukey’s pairwise comparison. No significant results were obtained in the KP-N-YN model.


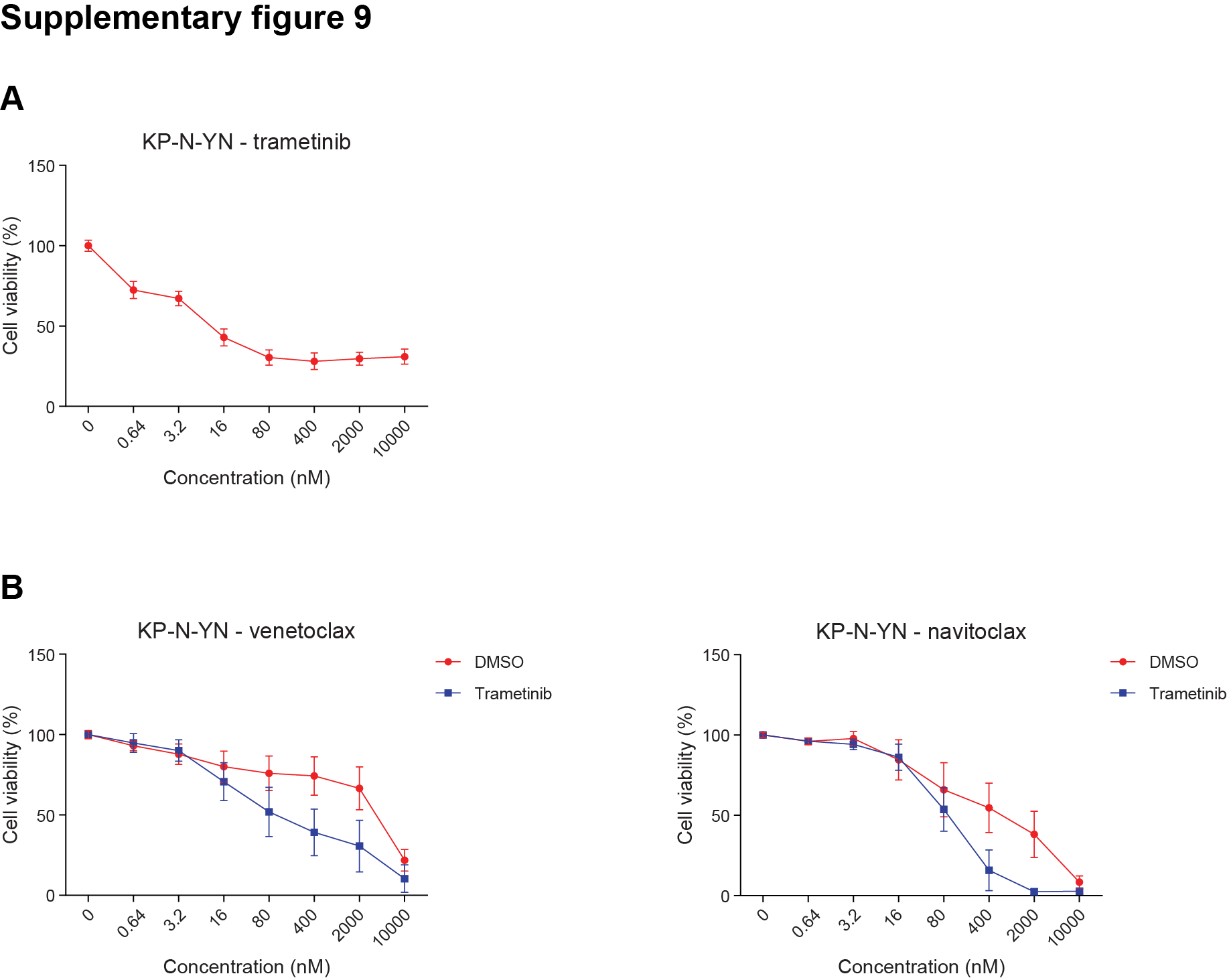


**Supplementary Figure 11:** *In vitro* effects of trametinib monotherapy and combination therapy with BCL-2 inhibitors on KP-N-YN neuroblastoma cells. **(A)** Cell viability curve of KP-N-YN cells treated with a concentration range of trametinib. **(B)** Cell viability curves of KP-N-YN cells treated with venetoclax or navitoclax in the presence and absence of 1 µM trametinib. Values were normalized to trametinib- and DMSO-treated cells, respectively.
